# From behavioral context to receptors: serotonergic modulatory pathways in the IC

**DOI:** 10.3389/fncir.2012.00058

**Published:** 2012-09-06

**Authors:** Laura M. Hurley, Megan R. Sullivan

**Affiliations:** Department of Biology, Center for the Integrative Study of Animal Behavior, Indiana UniversityBloomington, IN, USA

**Keywords:** serotonin, receptor, 5-HT1A, 5-HT1B, 5-HT2, 5-HT3, behavioral context

## Abstract

In addition to ascending, descending, and lateral auditory projections, inputs extrinsic to the auditory system also influence neural processing in the inferior colliculus (IC). These types of inputs often have an important role in signaling salient factors such as behavioral context or internal state. One route for such extrinsic information is through centralized neuromodulatory networks like the serotonergic system. Serotonergic inputs to the IC originate from centralized raphe nuclei, release serotonin in the IC, and activate serotonin receptors expressed by auditory neurons. Different types of serotonin receptors act as parallel pathways regulating specific features of circuitry within the IC. This results from variation in subcellular localizations and effector pathways of different receptors, which consequently influence auditory responses in distinct ways. Serotonin receptors may regulate GABAergic inhibition, influence response gain, alter spike timing, or have effects that are dependent on the level of activity. Serotonin receptor types additionally interact in nonadditive ways to produce distinct combinatorial effects. This array of effects of serotonin is likely to depend on behavioral context, since the levels of serotonin in the IC transiently increase during behavioral events including stressful situations and social interaction. These studies support a broad model of serotonin receptors as a link between behavioral context and reconfiguration of circuitry in the IC, and the resulting possibility that plasticity at the level of specific receptor types could alter the relationship between context and circuit function.

## Introduction

The convergence of ascending, descending, and lateral circuitry in the inferior colliculus (IC) creates neural response properties important for decoding specific features of the auditory scene (Huffman and Henson, 1990; Vater et al., 1995; Kidd and Kelly, 1996; Thompson and Schofield, 2000; Malmierca et al., 2005a,b, 2009; Schofield and Coomes, 2006; Winer, 2006; Yavuzoglu et al., 2010; Oertel et al., 2011; Yavuzoglu et al., 2011). To build circuits that respond to changing behavioral contexts, information that is extrinsic to the auditory system must also be incorporated into this convergence. Inputs signaling behavioral context or internal state strongly influence the responses of neurons in the IC to auditory stimuli. For example, external cues indicating the presence of a social partner, or internal signals of stress or reproductive state, can alter evoked responses or gene expression in the IC and its homologs in nonmammalian vertebrates (Mello and Ribeiro, 1998; Miranda and Wilczynski, 2009a,b; Mazurek et al., 2010; Mangiamele and Burmeister, 2011). There are numerous mechanisms through which extra-auditory sources of information influence neural processing in the IC, including input pathways from visual and somatosensory systems or pathways from affective centers such as the amygdala (Groh et al., 2001; Marsh et al., 2002; Metzger et al., 2006; Zhou and Shore, 2006; Dehmel et al., 2008). Hormonal pathways (Greco et al., 1999; Charitidi and Canlon, 2010; Maney and Pinaud, 2011) and centralized neurochemical pathways (Levitt and Moore, 1979; Klepper and Herbert, 1991; Thompson et al., 1994; Habbicht and Vater, 1996; Wynne and Robertson, 1996; Kaiser and Covey, 1997; Tong et al., 2005; Ji and Suga, 2009; Motts and Schofield, 2009, 2011) also provide potential routes for nonauditory signals.

Neuromodulatory networks comprise a large class of mechanisms linking behavioral context and internal state to local changes in auditory processing within the IC. Multiple neuromodulators including norepinephrine, acetylcholine, serotonin, and dopamine are present in the IC (Levitt and Moore, 1979; Klepper and Herbert, 1991; Thompson et al., 1994; Habbicht and Vater, 1996; Wynne and Robertson, 1996; Kaiser and Covey, 1997; Tong et al., 2005; Ji and Suga, 2009; Motts and Schofield, 2009, 2011). Neurons synthesizing these modulatory signals typically respond to salient features of behavioral situations and communicate this information to the IC through axonal projections that may ramify widely in the IC and other regions of the central nervous system. Although in some cases auditory neurons contain these neurochemicals, they can also be found in centralized extra-auditory nuclei (Klepper and Herbert, 1991; Tong et al., 2005; Motts and Schofield, 2009). Neuromodulatory systems have the ability to profoundly alter auditory processing in the IC, facilitating signatures of associative and other types of plasticity, or altering excitatory-inhibitory interactions to change ongoing responses to stimuli (Farley et al., 1983; Habbicht and Vater, 1996; Yigit et al., 2003; Hurley et al., 2004; Ji and Suga, 2009).

This review will focus on how the serotonergic neuromodulatory system in particular alters the function of circuits in the IC. The first reason for this focus is that the serotonergic system is unambiguously extrinsic to the auditory system in adults (Klepper and Herbert, 1991; Thompson and Lauder, 2005; Thompson, 2006; Thompson and Thompson, 2009). The serotonergic system is thus a clear candidate source of nonauditory information to the IC. Second, the serotonergic system is potentially a conduit for several major classes of nonauditory information to the IC, including information on the sleep-wake cycle, stress, and social encounters or social status (Boutelle et al., 1990; Mas et al., 1995; Clement et al., 1998; Jacobs and Fornal, 1999; Portas et al., 2000). Lastly, serotonin strongly modulates the sound-evoked activity of IC neurons (Hurley and Pollak, 1999, 2001; Hurley et al., 2002).

### Sources of serotonin are extrinsic to the IC

**Figure 1:**
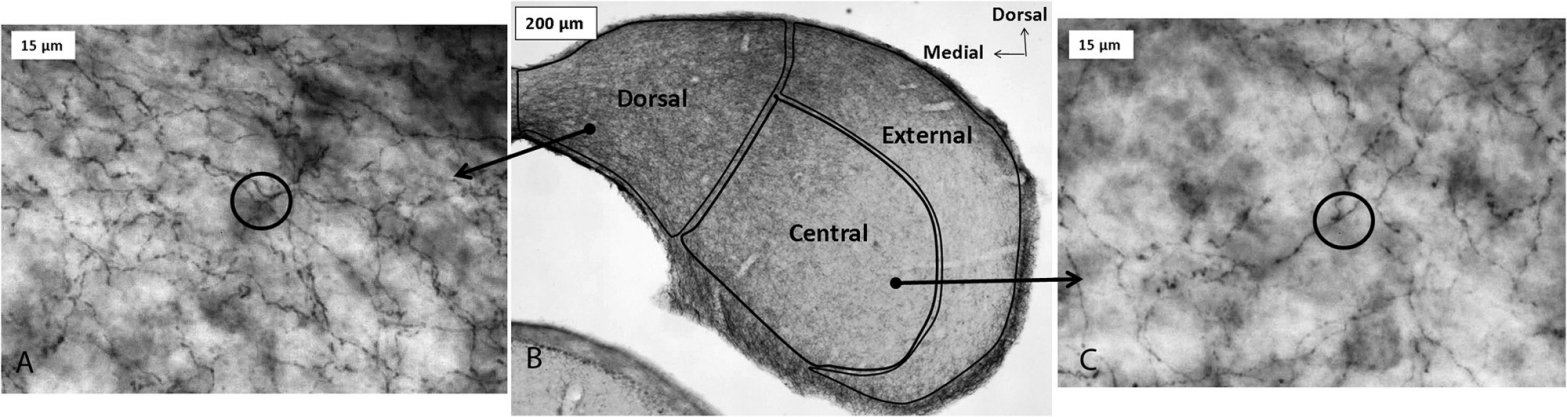
**Immunoreactivity for the serotonin transporter (SERT) in the IC of a CBA/J mouse, showing projections from raphe nuclei.** Panel **(B)** contains a photomicrograph of a transverse section of IC processed with a SERT antibody, and panels **(A)** and **(C)** show higher-magnification images of positively immunostained fibers in the dorsal cortex and central IC, respectively. Circles in panels **(A)** and **(C)** depict software probes used to estimate fiber density. All panels were reprinted with permission from Elsevier; additional information on the source article can be found in the Acknowledgments.

Serotonergic innervation of the IC has been noted for decades. In adults, no auditory neurons contain serotonin, although during development neurons in some brainstem auditory nuclei are transiently immunopositive for the serotonin transporter (SERT) and accumulate serotonin (Thompson and Lauder, 2005; Thompson, 2006; Thompson and Thompson, 2009). Dense innervation of the auditory midbrain by serotonergic fibers is conserved across vertebrate groups, as is the relative density of fibers in different subnuclei (Figure 1; Klepper and Herbert, 1991; Kaiser and Covey, 1997; Zeng et al., 2007). Fibers are denser in shell regions such as the dorsal and external cortices, something that has been confirmed quantitatively (Klepper and Herbert, 1991; Thompson et al., 1994; Kaiser and Covey, 1997; Hurley and Thompson, 2001; Zeng et al., 2007; Matragrano et al., 2012; Papesh and Hurley, 2012). Although serotonergic projections are somewhat denser in cortical IC regions, substantial innervation by serotonergic fibers also occurs in the central subnucleus (Papesh and Hurley, 2012). Once released, serotonin may act either synaptically or extrasynaptically in some brain regions in a process known as volume release (Bunin and Wightman, 1999). A synaptic morphology suggestive of nonsynaptic release has been described for specialized serotonergic “basket terminals” found around a subset of GABAergic neurons in primary auditory cortex of cat (Defelipe et al., 1991). The structure of serotonergic release sites has not been explored in the IC, however.

The major source of serotonergic fibers to the IC is the dorsal raphe nucleus (DRN), a superior nucleus in a chain of serotonin-producing raphe nuclei in the vertebrate brain. Approximately 80% of raphe neurons that both contain an IC-injected retrograde tracer and co-label with an antibody to serotonin are found in the DRN, with smaller proportions found in other regions such as the median raphe nucleus (14%) (Klepper and Herbert, 1991). Beyond this simple characterization, little information is available regarding the significance of anatomical relationships between the raphe nuclei and the IC. For example, whether subgroups of DRN neurons disproportionately innervate the IC has not been established.

### The serotonergic system provides information on external events and internal state to the IC

Supporting the concept that the anatomical connection between the DRN and IC serves as a conduit for multiple types of nonauditory information into the IC, the DRN itself receives inputs from many other brain regions. These include relatively peripheral sensory areas such as the retina, vestibular nuclei, and purported multisensory regions that likely process sound (Kawano et al., 1996; Ye and Kim, 2001; Cuccurazzu and Halberstadt, 2008). They also include regions such as amygdala, preoptic areas, and prefrontal cortex (Peyron et al., 1998; Lee et al., 2003). As a consequence of these projections, the activity of DRN neurons is influenced by a wide range of behaviorally relevant factors. The tonic level of activity of DRN neurons depends heavily on the phase of the sleep-wake cycle, with firing rates higher in waking states. DRN neurons also respond transiently to simple sensory stimuli (Heym et al., 1982; Jacobs and Fornal, 1999), and follow higher-level features of behavioral paradigms such as the delay of a reward (Miyazaki et al., 2011).

**Figure 2:**
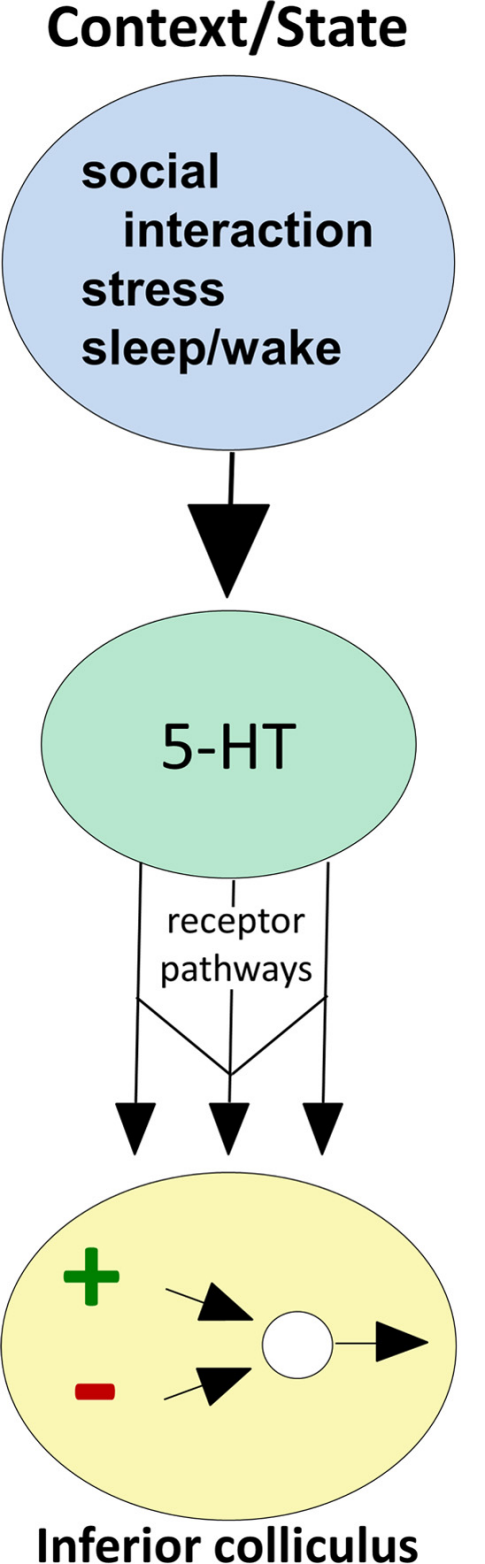
**Conceptual diagram illustrating how behavioral state modulates the function of circuitry in the IC, through serotonin receptor pathways.** Behavioral factors (top oval) evoke serotonin release (middle oval), which then acts through multiple receptor types expressed by IC neurons (arrows) to influence excitatory-inhibitory circuitry (bottom oval).

There is also direct evidence that both behavioral state and the occurrence of external events regulate serotonin levels within the IC (Figure 2; reviewed in Hurley and Hall, 2011). As expected from the influence of the sleep/wake cycle on activity in the DRN, locally measured serotonin in the IC increases as mice wake from anesthesia (Hall et al., 2010). On top of this tonic change, serotonin increases within minutes in the IC in response to loud broadband noise and to restriction stress, but not to other simple stimuli that evoke strong behavioral responses such as the presentation of food or odor of a predator (Hall et al., 2010). Serotonin also increases in the IC over the course of social interactions including same-sex resident-intruder interactions (Hall et al., 2011) and opposite-sex encounters (unpublished data). The dynamics of serotonergic increases are distinct across different types of behavioral paradigms, such that increases during social interactions are more gradual than during the presentation of simple stressors (Hurley and Hall, 2011).

### Serotonin strongly modulates auditory responses in the IC

The local application of exogenous serotonin and the release of local stores of serotonin have strong effects on the auditory responses of single IC neurons, supporting the idea that context-specific changes in serotonin have functional consequences (Hurley and Pollak, 1999; Hall and Hurley, 2007). A prominent effect of serotonin is to control the gain of IC responses across the frequency tuning curve. In most cases, serotonin suppresses responses evoked by tones and FM sweeps, but in a minority of neurons, serotonin facilitates responses (Hurley and Pollak, 1999, 2001). Among the more interesting effects of serotonin observed in a subset of IC neurons are frequency-specific changes in firing rate. For these neurons, serotonin may strongly suppress or facilitate responses to tones at some frequencies, but leave responses to other frequencies in the tuning curve relatively unaffected (Hurley and Pollak, 2001). Either uniform gain control or frequency-specific effects can result in changes in the bandwidths of frequency response ranges (Hurley and Pollak, 2001). Such changes in tuning further result in changes in the selectivity of neurons for more complex sounds.

Not surprisingly, the pronounced changes in spike rate induced by serotonin are often accompanied by changes in multiple temporal features of spike trains, including initial spike latency and variance, interspike interval (ISI), and the response durations of IC neurons (Hurley and Pollak, 2005b). Changes in temporal response properties such as the initial spike latency often correspond to changes in spike rate, so that spike suppression is associated with increased latency, and spike facilitation with decreased latency, but can also occur independently of changes in spike rate. In addition to changing temporal response properties of IC neurons, serotonin also targets neurons with particular temporal characteristics. Across multiple studies, neurons with latencies of over approximately 10 ms, or with relatively low first-spike precision, show greater sensitivity to serotonergic agonists including serotonin, a 5-HT1A agonist, and a 5-HT3 agonist (Hurley and Pollak, 2005b; Hurley, 2007; Bohorquez and Hurley, 2009). These consistent findings suggest that longer-latency or lower-precision neurons either preferentially express some types of serotonin receptors or are more responsive to activation of these receptors. Interestingly, IC neurons with latencies of over 12 ms are also more reliably sensitive to NMDA receptor blockade and less sensitive to AMPA receptor blockade (Sanchez et al., 2007). Together, these studies hint at the existence of distinct sets of neurochemical sensitivities in groups of IC neurons defined by their temporal characteristics.

Studies at the level of single IC neurons thus illustrate that serotonin and its receptors are capable of broadly reshaping temporal features of evoked activity in the IC. In agreement with this hypothesis, brain-wide depletion of serotonin using parachlorophenylalanine (pCPA) alters the latencies of auditory brainstem response (ABR) waves including wave V, which is likely to be influenced by activity in the IC (Funai and Funasaka, 1983; Kaga et al., 1999). The effect of pCPA may depend on the species under study or on stimulus parameters such as repetition rate, since the same type of serotonergic depletion has been reported to either decrease or increase ABR latencies (Revelis et al., 1998). Taken as a whole, however, these findings underscore the global effects of the manipulation of endogenous sources of serotonin on the timing of auditory activity.

## Serotonin receptor types define parallel pathways for regulation of IC circuitry

The strength and dynamics of the effects of endogenously released serotonin are regulated by local mechanisms in the IC, including high-affinity transporters expressed by serotonergic projections as well as lower-affinity organic cation transporters (Hoffman et al., 1998; Gasser et al., 2009; Hall et al., 2010). A particularly important aspect of local regulation of serotonergic effects is the expression of serotonin receptors by auditory neurons in the IC (Thompson et al., 1994; Hurley, 2006). Of all the classes of molecules regulating serotonergic signaling locally, different types of serotonin receptor have the most potential to provide insight into the wide range of serotonergic effects on IC neurons. This is because receptors are differentially expressed by IC neurons, and different receptor types have highly characteristic effector pathways and patterns of subcellular localization (Hannon and Hoyer, 2008). This diversity creates a wide range of potential neuromodulatory effects that are quite specific for the auditory circuitry of the IC, even though they originate from a diffusely projecting neurochemical system. Different serotonin receptor types therefore act as parallel pathways for altering specific features of circuitry in the IC (Figure 2).

Different serotonin receptor types represent an especially rich source of potential functional variability relative to receptors of other types of neuromodulators, because of the exceptionally wide array of serotonin receptor types. There are seven major families of serotonin receptors, several of which include multiple well-characterized and functionally distinct members (Hannon and Hoyer, 2008). Most of these families consist of G-protein coupled receptors. 5-HT3 receptors are the exception, and are cation channels similar in structure to nicotinic acetylcholine receptors (Chameau and Van Hooft, 2006). Consistent with this predominantly metabotropic set of effector pathways, the actions of serotonin receptors in the IC conform to classical models of neuromodulator function. That is, rather than directly creating responses to auditory stimuli, serotonin receptors reconfigure auditory circuits mediated by other neurotransmitters (Harris-Warrick, 2011). Although members of at least five families of serotonin receptor have been reported in the IC, members of only three of these have been explored in terms of their physiological function: 5-HT1, 5-HT2, and 5-HT3 receptors (Chalmers and Watson, 1991; Pompeiano et al., 1992; Thompson et al., 1994; To et al., 1995; Wright et al., 1995; Waeber et al., 1996; Morales and Bloom, 1997; Peruzzi and Dut, 2004; Hurley, 2006; Wang et al., 2008; Bohorquez and Hurley, 2009; Miko and Sanes, 2009).

**Figure 3:**
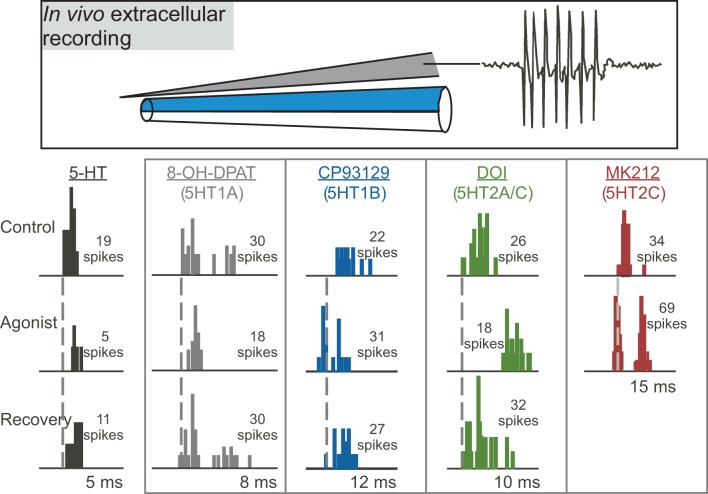
**Effects of different serotonin (5-HT) receptor agonists on spike number and latency in response to 32 repetitions of a tone at characteristic frequency.** The top panel depicts a “piggyback” electrode used for combined extracellular recording of single neurons and drug iontophoresis. The lower panel shows PSTHs of responses in the control, during the iontophoresis of agonists, and in the recovery are shown for five different sample neurons in the IC. Specific agonists and the receptors they target appear above each neuron. Dashed vertical lines mark the latencies of the earliest spikes in the control condition. Stimuli were: for serotonin, a 10-kHz FM sweep centered at 25 kHz at 20 dB SPL; for 8-OH-DPAT, a 10-kHz FM sweep centered at 25 kHz at 40 dB SPL; for CP93129, a 20-kHz tone at 20 dB SPL; for DOI, a 7-kHz FM sweep centered at 21 kHz at 50 dB SPL; for MK212, a 5-kHz FM sweep centered at 19 kHz at 50 dB SPL. All stimuli were 10 ms in duration. The lower panel was reprinted from a journal published by the American Physiological Society; additional information on the source article can be found in the Acknowledgments.

Multiple studies on the function of specific serotonin receptor types in the IC have used the approach of extracellular recording of sound-evoked responses *in vivo* during the local iontophoresis of serotonin receptor agonists and antagonists (Figure 3; Hurley and Pollak, 1999, 2001, 2005a,b; Hurley, 2006, 2007; Hurley et al., 2008; Bohorquez and Hurley, 2009; Ramsey et al., 2010). In these types of studies, inferences on the influence of serotonin receptor types on particular features of the circuitry of the IC are based on three types of information: the demonstrated cellular mechanisms of these receptors throughout the brain, their specific effects in the IC, and their interaction with neurotransmitter systems in the IC. With several important exceptions that are described below, the cellular mechanisms of serotonergic action in the IC are therefore largely unexplored through *in vitro* recording techniques. In the remainder of this section, we present direct and indirect evidence supporting roles for specific serotonin receptor pathways in the modulation of inhibition, the suppression of responsiveness to input, and the mediation of activity-dependent plasticity. The interaction of some serotonin receptor types is also described.

### Serotonergic modulation of inhibition

The IC contains a strong inhibitory network and excitatory-inhibitory balance is particularly important for acoustic processing in the IC (Yang et al., 1992; Casseday et al., 1994; Fuzessery and Hall, 1996; Lebeau et al., 1996; Casseday et al., 2000). Serotonergic modulation of inhibition is one type of mechanism that shifts the excitatory-inhibitory balance and selectively tunes sensory processing (Ciranna, 2006). GABA is a major inhibitory neurotransmitter in the IC and activates two types of receptors, GABA_A_ and GABA_B_ receptors (Wisden et al., 1992; Fubara et al., 1996). GABA_A_ receptors directly pass Cl^−^ and have modulatory binding sites for benzodiazepines, barbiturates, neurosteroids, and ethanol (Macdonald and Olsen, 1994). In contrast, GABA_B_ receptors couple to Ca^2+^ and K^+^ channels via G proteins and second messengers (Bowery, 1989). Serotonergic inputs could also potentially modulate glycinergic inhibitory transmission, but there is currently little data on the effects of serotonin on glycinergic inhibition in the IC or other brain areas (Engelhardt et al., 2010). For this reason, we will focus on serotonergic modulation of GABAergic transmission by describing (1) the anatomical association between the serotonergic and GABAergic systems, and (2) the modulation of GABAergic transmission by serotonin.

#### Anatomical association between 5-HT receptors and inhibitory interneurons

The serotonergic nucleus providing most of the serotonergic fibers to the IC, the DRN, has a distinct relationship with GABAergic neurons in other brain regions that provides a useful comparison for the IC. In the hippocampus and cortex, selective serotonergic modulation of inhibitory neurons has been observed (Freund et al., 1990; Defelipe et al., 1991). In particular, the serotonergic raphe-hippocampal pathway forms multiple synaptic contacts with calbindin D-28k-containing GABAergic interneurons, but not parvalbumin-expressing GABAergic interneurons (Freund et al., 1990). Both calbindin and parvalbumin are calcium binding proteins usually located in inhibitory interneurons. This suggests that the serotonergic pathway may influence synaptic function through modulation of local inhibitory circuits. Similar anatomical targeting of projections from the DRN onto GABAergic neurons has been observed around somata and dendrites of GABAergic neurons in primary auditory cortex (Defelipe et al., 1991). In other cortical areas, inhibitory interneurons have also been identified as major targets of serotonergic synapses (Smiley and Goldmanrakic, 1996). This input specificity from raphe axons suggests a modulatory diversity in inhibitory cell types.

In the IC, it is not known whether serotonergic inputs to the IC target specific inhibitory neuron cell types as observed in the hippocampus and cortex. However, staining for both calbindin and parvalbumin, which label neuronal cell types targeted by raphe projections in the hippocampus and cortex, is localized to the superficial rim of the external cortex and dorsal cortex and is almost completely absent from the central nucleus (Celio, 1990; Lohmann and Friauf, 1996). To some extent, the distribution of these presumptive inhibitory interneurons therefore parallels the density of serotonergic fibers in the subregions of the IC.

In addition to targeting at the level of serotonergic projections, there is evidence for anatomical segregation of serotonergic effects based on the selective expression of serotonin receptors by different cell types (Jakab and Goldman-Rakic, 2000). In cortical neurons, 5-HT2A receptors are segregated from 5-HT3 receptors. 5-HT2A receptors are found in pyramidal neurons and in GABAergic interneurons expressing parvalbumin and calbindin. 5-HT3 receptors are found in small GABAergic interneurons with calbindin and medium calretinin-containing interneurons (Morales and Bloom, 1997; Jakab and Goldman-Rakic, 2000). This cellular segregation indicates a serotonin-receptor specific segmentation of the GABAergic inhibitory actions along the pyramidal neuronal arbor (Jakab and Goldman-Rakic, 2000).

There is some evidence for the expression of serotonin receptor types by GABAergic neurons in the IC. Approximately two-thirds of GABA-positive neurons are associated with 5-HT1A or 5-HT1B receptors, with neurons positive for 5HT1B receptors more numerous than neurons positive for 5HT1A receptors (Peruzzi and Dut, 2004). GABAergic neurons in the IC can additionally be subdivided into at least two groups based on soma size and their innervation by different types of glutamatergic terminals (Ito et al., 2009), but whether serotonin differentially regulates these neural classes is unknown. This association between serotonin receptors and GABAergic neurons provides some suggestive evidence that serotonergic modulation may target the inhibitory network in the IC, similar to mechanisms in other brain regions (Peruzzi and Dut, 2004).

It is essential to note that IC neurons receive GABAergic inputs from a range of sources. The dorsal nucleus of the lateral lemniscus (DNLL) sends a bilateral GABAergic projection to IC that shapes binaural responses (Adams and Mugnaini, 1984; Shneiderman et al., 1988; Shneiderman and Oliver, 1989; Faingold et al., 1993; Shneiderman et al., 1993; Burger and Pollak, 2001; Pollak et al., 2002), and other nuclei of the lateral lemniscus also contain GABAergic neurons that project to IC (Zhang et al., 1998). GABAergic projections to IC also arise from neurons in the contralateral IC (Gonzalez-Hernandez et al., 1996; Hernandez et al., 2006). Small numbers of GABAergic neurons project to the IC from periolivary regions and cochlear nuclei (Gonzalez-Hernandez et al., 1996). IC neurons also receive projections from intrinsic GABAergic neurons, which constitute a substantial proportion of cell bodies in the IC (Roberts and Ribak, 1987; Oliver et al., 1994). Which of these populations of GABAergic neurons are modulated by the 5-HT1B and 5-HT2A receptors has not yet been explored, but this information could provide an important clue as to the functional consequences of the modulation of inhibition by serotonin receptors.

#### Modulation of GABAergic inhibition

Multiple studies have explored the functional interaction between serotonin receptors and GABAergic inhibition. *In vitro* evidence from one study suggests that the 5-HT2A receptor modulates GABAergic inhibition in the IC (Wang et al., 2008). In this study, serotonin enhanced the frequency and amplitude of spontaneous inhibitory postsynaptic currents (sIPSCs) in 70% of neurons recorded. A selective 5-HT2A agonist (α-methylserotonin) mimicked the effect of serotonin on sIPSCS. The observation of enhanced sIPSCs in the IC is consistent with data from other brain regions including the substantia nigra (Stanford and Lacey, 1996) and cortex (Zhou and Hablitz, 1999).

Additional support for a role of the 5-HT2 receptor dependent modulation of inhibition in the lateral superior olive (LSO) is supported by a series of experiments using whole cell voltage-clamp recordings of gerbil slices from postnatal days 6–13 (Fitzgerald and Sanes, 1999). These experiments identified an increase in spontaneous inhibitory postsynaptic currents (IPSCs) with the application of 5-HT. Upon further examination, a 5-HT2 agonist (α-Me-5-HT) reproduced the increase in spontaneous IPSCs and a 5-HT2 antagonist (ketanserin) blocked the induction of spontaneous IPSCs. This specific modulation of inhibition by the 5-HT2 receptor was developmentally dependent and generally not observed beyond postnatal day 8. It is not yet known if a similar developmentally dependent mechanism of serotonergic modulation might be observed in the IC or other regions in the auditory pathway.

**Figure 4:**
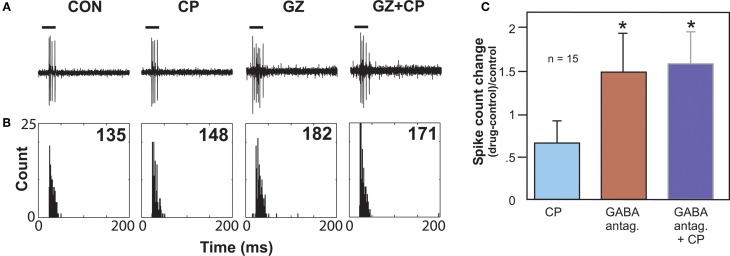
**Effects of GABA_A_ antagonists alone and with 5-HT1B agonist CP93129. (A)** Single voltage traces in response to a CF pure tone of 11 kHz for each drug condition. **(B)** PSTH plots consisting of 32 trials with a 250 ms duration for each trial and corresponding to the voltage traces in **(A)**. Numbers represent total spike counts for the 32 tone presentations. Both CP93129 (CP) and the GABA_A_ antagonist gabazine (GZ) increased spikes alone, but CP93129 did not increase spikes in the presence of gabazine (GZ + CP) relative to gabazine alone. **(C)** Average changes in spike count at CF relative to the control condition for 15 neurons. Panel **(B)** was reprinted from a journal published by the American Physiological Society; additional information on the source article can be found in the Acknowledgments.

Another type of serotonin receptor, the 5-HT1B receptor, may also affect GABAergic transmission. Activation of this receptor type strongly facilitates sound-evoked responses (Hurley, 2006). In other brain regions, the 5-HT1B receptor is localized to presynaptic axons or terminals and decreases the release of a range of neurotransmitters (Sari, 2004). These facts underlie the hypothesis that the 5-HT1B receptor decreases the release of inhibitory neurotransmitter in the IC. An *in vivo* study examined whether the 5-HT1B receptor influences responses to GABAergic inhibition in the IC by using the selective 5-HT1B agonist CP93129 in combination with the co-application of GABA_A_ receptor antagonists (bicuculline or gabazine; Figure 4). This combination tested the prediction that 5-HT1B activation and blockade of GABAergic inhibition would create similar effects. Indeed, 5-HT1B activation and GABA_A_ blockage, when each manipulation was performed alone, had similar effects on the direction and magnitude of responses, frequency response bandwidths, and ISIs, although the effects of GABA_A_ blockade were typically larger. Furthermore, following the increase in spike number induced by GABA_A_ receptor antagonists alone, the addition of a 5-HT1B agonist did not cause a further facilitation, suggesting that blockage of GABA_A_ receptors reduced the effect of activating 5-HT1B receptors (Hurley et al., 2008). Taken together with the localization of 5-HT1B receptors to presynaptic axons or terminals in other brain regions, these experiments provide evidence for modulation of GABAergic inhibition in the IC by the 5-HT1B receptor.

Multiple authors have found that GABAergic inhibition surrounding the characteristic frequencies of IC neurons sharpens frequency tuning (Yang et al., 1992; Fuzessery and Hall, 1996; Palombi and Caspary, 1996; Lebeau et al., 2001; Lu and Jen, 2001). A functional test of whether the 5-HT1B receptor regulates excitatory-inhibitory balance in these frequency regions was performed *in vivo* by suppressing the excitatory responses to a tone at the characteristic frequency with the co-presentation of tones at surrounding frequencies (Hurley et al., 2008). The effect of a 5-HT1B selective agonist (CP93129) on the excitatory-inhibitory balance was then tested using such two-tone stimuli. The 5-HT1B agonist often decreased the suppression of the tone at characteristic frequency by tones at surrounding frequencies (Hurley et al., 2008). The 5-HT1B agonist also expanded frequency tuning in many neurons, and in some cases the frequencies of expansion corresponded to frequencies at which reduction of suppression occurred. This suggests that the 5-HT1B receptor regulates the sharpness of frequency tuning in some IC neurons by reducing surround inhibition.

All of the studies described above indicate that GABAergic inhibition in the IC is regulated by multiple types of serotonin receptor, which influence GABAergic transmission in different ways.

### Suppression of responses by serotonin

A common effect of applying serotonin is the suppression, or decrease in magnitude, of evoked responses and spontaneous activity in IC neurons (Hurley and Pollak, 1999). Suppression by serotonin could be achieved through multiple serotonin receptor types and through pre- and postsynaptic mechanisms. The 5-HT1A receptor is likely to be one of the receptors, if not the predominant receptor, mediating suppression of responses. The expression of the 5-HT1A receptor is widespread throughout the IC including expression in inhibitory neurons (Duncan et al., 1998; Peruzzi and Dut, 2004). The 5-HT1A receptor is often located somatodendritically and typically decreases postsynaptic responses through an association with inward-rectifying potassium channels (Okuhara and Beck, 1994; Polter and Li, 2010).

**Figure 5:**
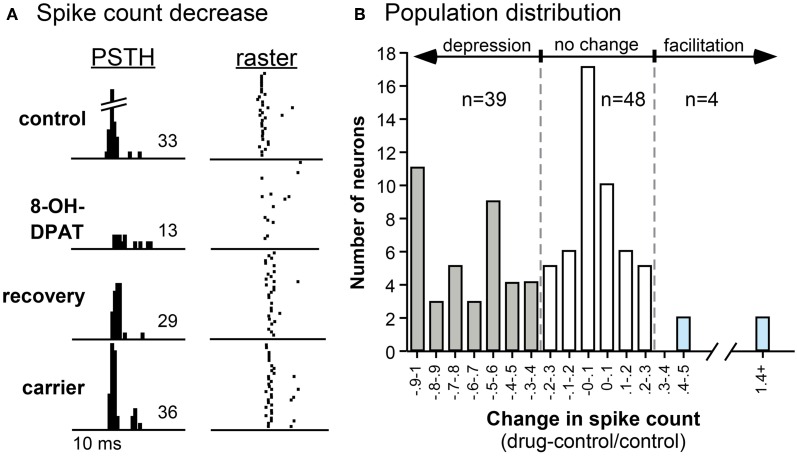
**Effects of the selective 5-HT1A agonist 8-OH-DPAT on a single neuron (A) and on a population of IC neurons (B). (A)** PSTH and raster plots of the response of a neuron to an FM sweep in the control, during iontophoresis of 8-OH-DPAT, during the recovery, and during the iontophoresis of the carrier solution. The stimulus was a 30-dB SPL, 10-kHz FM sweep centered at 25 kHz. **(B)** Histogram of changes in spike count evoked by 8-OH-DPAT in 91 IC neurons. Negative values indicate decreases in spike count relative to the pre-drug control, and positive values indicate increases in spike count. Across the neuron population, the effect of 8-OH-DPAT is predominantly suppressive. All panels were reprinted with permission from Elsevier; additional information on the source article can be found in the Acknowledgments.

Experiments in the IC of the mouse and Mexican free-tailed bat have shown that a 5-HT1A receptor agonist (8-OH-DPAT) suppress responses and shifts the temporal response profile to pure tones and FM sweeps (Figure 5; Hurley, 2006, 2007). 5-HT1A activation often increases the first-spike latency and ISI, while differentially suppressing secondary spikes in IC neurons (Hall and Hurley, 2007; Ramsey et al., 2010). Interestingly, the 5-HT1A receptor agonist produces the effects most similar to applying serotonin alone when compared to agonists for 5-HT1B and 5-HT2C receptors, suggesting that the 5-HT1A receptor may play a comparatively strong role in serotonergic modulation (Hurley, 2006).

Similar to the diversity of effects of serotonin (Hurley and Pollak, 1999), selective activation of the 5-HT1A receptor results in facilitation in a small number of IC neurons (Hurley, 2006). The net result of 5-HT1A receptor-induced hyperpolarization may depend on whether the receptors are present on an excitatory or inhibitory neuron. With an excitatory neuron, the result would be a direct suppression of responses. However, with an inhibitory neuron, the result could be facilitation of the responses of postsynaptic neurons. In several brain regions, the 5-HT1A receptor has been demonstrated to alter presynaptic GABA release (Kishimoto et al., 2001; Lee et al., 2008). Additional experiments in the IC with 5-HT1A agonists and antagonists in combination with GABA_A_ receptor antagonists (bicuculline or gabazine) will be required to determine the interactions between the 5-HT1A receptor and GABAergic inhibition in the IC.

A common consequence of suppression by the 5-HT1A receptor is a narrowing in the frequency tuning curves of the neurons. Functionally, response suppression may therefore serve to increase the selectivity of responses to auditory stimuli. This would provide an additional mechanism in addition to direct shifts in the inhibitory-excitatory synaptic balance for tuning of responses to auditory stimuli. A range of mechanisms, including suppression and inhibition, likely acts concurrently to result in behavioral context-related changes in the IC. Suppression based on postsynaptic hyperpolarization may provide an important mechanism for serotonergic-dependent tuning of auditory processing.

### Short-term plasticity and the 5-HT3 receptor

Of all serotonin receptor types in the IC, 5-HT3 receptors might be expected to mediate the most rapid and neurotransmitter-like effects. 5-HT3 receptors have a pentameric structure similar to that of nicotinic acetylcholine receptors (Chameau and Van Hooft, 2006). Also similar to AChRs, 5-HT3 receptors are cation channels that allow the passage of Na+ and Ca2+ ions. Consistent with this mechanism of action, activation of 5-HT3 receptors does create rapid effects in some IC neurons, in seconds or less, comparable to application of neurotransmitters such as GABA and glutamate (Bohorquez and Hurley, 2009). For many IC neurons, however, the effects of activating the 5-HT3 receptor are much more slowly developing, with half-maximal effects occurring over a range from 10 to more than 150 s, with correspondingly long recovery times. Furthermore, in a majority of 55% of neurons that respond to a 5-HT3 agonist, the effects of 5-HT3 activation are suppressive rather than excitatory. One possible mechanism that could account for a suppressive effect of the 5-HT3 receptor is that it excites presynaptic inhibitory neurons such as GABAergic neurons (Shneiderman et al., 1993; Oliver et al., 1994; Gonzalez-Hernandez et al., 1996; Zhang et al., 1998). GABAergic neurons are a target of the 5-HT3 receptor in other sensory systems, making this a plausible mechanism (Morales and Bloom, 1997; Xiang and Prince, 2003). This potential regulation of GABergic pathways by the 5-HT3 receptor was assessed in one study by comparing the effects of a 5-HT3 receptor agonist in the presence versus absence of a GABA_A_ antagonist. The rationale behind this experiment was similar to that used for assessing whether the 5-HT1B receptor interacted with GABAergic transmission, with the logic that blocking GABA_A_ receptors should reduce the effect of 5-HT3 activation, if the 5-HT3 receptor acts by increasing GABA release. Unlike the 5-HT1B receptor, the effect of 5-HT3 activation was not decreased and was even accentuated in some neurons in the presence of a GABA_A_ antagonist (Bohorquez and Hurley, 2009). This finding led to rejection of the hypothesis that the 5-HT3 receptor suppresses evoked activity in many IC neurons by exciting presynaptic GABAergic neurons.

A second hypothesis that could account for the range of effects of the 5-HT3 receptor in the IC is that this receptor has a genuinely modulatory influence via the admission of calcium into IC neurons. An interesting comparison between two independent studies examining the 5-HT3 receptor in the IC provides evidence for this hypothesis. One of these studies measured responses to current injection *in vitro* (Miko and Sanes, 2009). Following strong stimulation of lemniscal input fibers, the spiking response to a threshold-level current injection exhibited a short-term change in gain, usually facilitation, lasting seconds to minutes. Blockade of the 5-HT3 receptor removed or reduced the effect of fiber stimulation on gain control, at the same time reducing an associated depolarization of membrane potential. In contrast, antagonists of multiple other transmitter pathways, including ionotropic GABAergic and glycinergic receptors and both nicotinic and muscarinic cholinergic receptors, facilitated the change in gain. Furthermore, a similar decrease in positive gain was created by blockade of L-type calcium channels. These results all suggest an induction of plasticity in response gain by the 5-HT3 receptor, potentially by the postsynaptic admission of calcium (Miko and Sanes, 2009).

**Figure 6:**
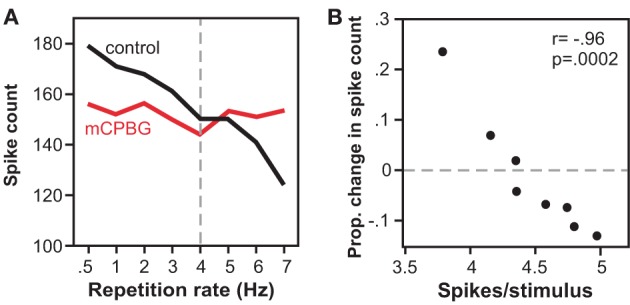
**Effects of the 5-HT3 receptor vary with stimulus repetition rate and spike rate. (A)** Spike counts decrease with repetition rate in the control, but not during iontophoresis of a 5-HT3 agonist (mCPBG) for a single IC neuron. Stimulus consisted of a 20 ms tone at 13 kHz. **(B)** The same data as in **(A)** is plotted as a function of mCPBG-evoked change in spike count versus control spike rate. The 5-HT3 agonist increases spike count at low spike rates, and decreases spike count at high spike rates. All panels were reprinted with permission from Elsevier; additional information on the source article can be found in the Acknowledgments.

In a companion study *in vivo*, the effect of activating the 5-HT3 receptor corresponded to the level of spiking (Bohorquez and Hurley, 2009). Across the IC neuron population, higher rates of evoked spiking were associated with larger effects of 5-HT3 receptor activation. In a subset of single neurons, the effect of 5-HT3 receptor activation varied depending on the stimulus repetition rate and consequent spike rate per stimulus (Figure 6). As the stimulus presentation rate increased, associated with a decrease in the spike number, the effect of the 5-HT3 receptor shifted from relative suppression to relative facilitation. Functionally, the 5-HT3 agonist flattened the relationship between stimulus presentation rate and spike rate, in effect equalizing neural responses across different repetition rates. Together, these *in vitro* and *in vivo* studies suggest that the 5-HT3 receptor underlies a form of short-term plasticity in the IC, altering response gain in an activity-dependent manner, and potentially acting to stabilize the level of activity of IC neurons to stimulus trains.

### Interactions of receptors create novel combinations of effects

As instructive as it is to understand the effects of individual serotonin receptor types, a realistic model of the effects of serotonin in the IC must involve some degree of receptor interaction, because multiple receptor types would likely be co-activated by the release of serotonin. With the diversity of receptor types in the IC, many of which have disparate cellular modes of action and distinct effects on evoked activity, predicting the nature of receptor interaction is not a straightforward matter. A study on the interactions of two specific serotonin receptor types within the same receptor family, the 5-HT1A and 5-HT1B receptor, illustrates this point.

The 5-HT1A and 5-HT1B receptors make an interesting comparison, since both are expressed widely among IC neurons (Thompson et al., 1994; Peruzzi and Dut, 2004), and activation of each receptor type correspondingly influences evoked responses of a large proportion of IC neurons. As described earlier, the effects of these receptors on evoked activity in response to tones are opposite, in that activation of the 5-HT1A receptor suppresses evoked responses and in some neurons reduces the bandwidth of frequency tuning, while activation of the 5-HT1B receptor facilitates evoked responses and often increases the bandwidth of frequency tuning. Each receptor also influences the timing of evoked responses for some neurons, with the 5-HT1A receptor usually increasing first-spike latency and ISI, and the 5-HT1B receptor often decreasing first-spike latency and ISI.

If they are not to simply antagonize each other, there are several potential ways for the 5-HT1A and 5-HT1B receptor to functionally interact. One of these is that they are segregated from each other on separate local circuits in the IC, so that they do not directly interact at all. In support of this, some IC neurons show disproportionately greater effects of activation of either the 5-HT1A or 5-HT1B receptors. Functional segregation of the two receptor types is not the complete solution to the puzzle of receptor interaction, however, because many IC neurons show substantial responses to individual activation of both receptor types (Ramsey et al., 2010). This suggests that both receptor types do influence the same local circuitry, as reflected in the activity of a single neuron. For these multiple-responsive neurons 5-HT1A and 5-HT1B receptors do not simply antagonize each other. When activated concurrently, an interesting differential effect of the two receptors types on spike rate versus spike timing emerges. For measures of evoked activity that reflect spike number, including spike rate at the characteristic frequency or the bandwidth of frequency tuning, the receptors do interact additively. That is, the effect of combinatorial activation of the two receptor types on spike rate can generally be predicted by summating the effect of each receptor type alone for a given neuron. In contrast, the 5-HT1A receptor dominates measures of spike timing including the first-spike latency and ISI, so that only the effect of the 5-HT1A receptor predicts the effect of the drug combination. These results fit the model of the 5-HT1A receptor as located postsynaptically on soma and dendrites, exerting control over the timing of spikes by causing potassium channels to open. These findings not only provide a resolution to the puzzle of receptor co-activation, but suggest that general suppression of responsiveness (5-HT1A) combined with presynaptic disinhibition (5-HT1B) can create novel aggregate effects, even for simple sounds such as tones at CF.

### Conclusions

**Figure 7:**
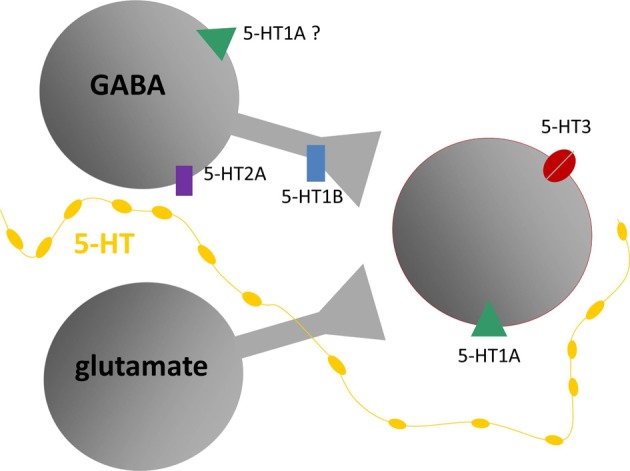
**Model of putative locations of 5-HT1A and 5-HT1B receptors relative to GABAergic and glutamatergic circuitry in the IC, based on *in vivo* and *in vitro* recordings.** Gray shapes are auditory neurons that express serotonin (5-HT) receptors responding to release of serotonin by projections from raphe nuclei (yellow line). Red outline indicates neurons recorded in the IC, receiving inputs from presynaptic GABAergic and glutamatergic neurons. Diagram does not include all known types of serotonin receptors or types of inputs received by IC neurons.

The experiments described above support the model of different serotonin receptor types as gates to separate but interacting effector pathways. As such, they are a critical node gating the route from behavioral context to the function of the circuitry of the IC. Table 1 and Figure 7 summarize the outcomes of studies on different receptor types in the IC and present a simplified model of the role of these receptors in the excitatory-inhibitory circuitry of the IC. Many features of this model, such as whether particular receptor types play special roles in specific contexts by virtue of their dynamics or closeness to serotonin release sites, are still murky. Nevertheless, the model provides a key for specific predictions in two broad areas of interest in understanding auditory function: (1) the role of the serotonergic system in specific behavioral contexts such as social interaction, and (2) how plasticity in the serotonergic system could alter the relationship between behavioral context and auditory processing or perception.

**Table 1:** **Summary of the effects of different serotonin receptor types in the IC**.

**5-HT receptor type**	**Localization**	**Preparation**	**Effects in IC**
1A	somatodendritic^1^	*in vivo*^a^,^b^	decreases evoked responses
1B	axonal/terminal^2^	*in vivo*^a^,^c^	increases evoked responses
2A	variable^3^	*in vitro*^d^	increases frequency and amplitude of GABAergic IPSCs
2C	postsynaptic density, somatodendritic^4^	*in vivo*^a^	increases evoked responses
2A/C	–	*in vivo*^a^	decreases evoked responses
3	variable^5^	*in vivo*, *in vitro*^e^	mixed gain control, activity-dependent

1 Hannon and Hoyer, 2008;

2 Sari, 2004;

3 Bombardi, 2012; Jakab and Goldman-Rakic, 1998;

4 Anastasio et al., 2010; Liu et al., 2007;

5 Carrillo et al., 2010;

a Hurley, 2006;

b Hurley, 2007;

c Hurley et al., 2008;

d Wang et al., 2008;

e Miko and Sanes, 2009; Bohorquez and Hurley, 2009.

## Context-dependence of serotonergic regulation

**Figure 8:**
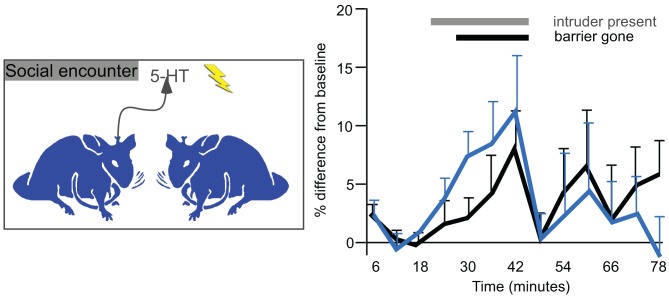
**Voltammetrically measured serotonin in the IC in resident male mice presented with a novel conspecific intruder (left panel).** The right panel plots changes in serotonin over baseline measurements. The gray bar represents time in which an intruder male was placed in the cage of a resident. The black bar represents time in which a Plexiglass barrier was removed and males directly interacted. The black line represents the initial interactions, and the blue line represents interactions with a new intruder by the same residents one week later. Serotonin increases gradually during the social interaction, and declines abruptly afterwards. The right panel was reprinted from a journal published by the American Psychological Association; additional information on the source article can be found in the Acknowledgments.

The measurement of serotonin in the IC of behaving animals suggests that serotonin regulates auditory processing in multiple behavioral contexts, including in waking states, in response to stressful or potentially threatening events, and during social interactions. Changes in serotonin that occur during the latter are worth examining in some detail, since they track behaviorally salient aspects of social interactions such as individual variation in social responses, experience, and age. In mice in which serotonin has been measured in the IC using voltammetry, individual differences in the serotonergic response to a novel social partner are quite pronounced, and correspond to social behaviors such as anogenital investigation and to nonsocial behaviors such as the overall level of activity (Hall et al., 2011). These behavioral correlations suggest that the serotonergic signal in the IC conveys information about the responsiveness of individual mice to a social stimulus. Moreover, being in a social experiment leaves a long-lasting trace, since both the serotonergic signal in the IC and some behaviors are increased in male mice undergoing a second social encounter occurring one week later, although this could be an effect of either the social interaction itself or of stress (Figure 8). Finally, predictable factors like age also significantly correlate with not only the serotonergic response to a social encounter but also the density of serotonergic fibers, both of which decrease over a period of young adulthood (Hall et al., 2011). All of these results describe a regulatory signal that varies with socially salient information.

The IC and its homologs, including the torus semicircularis in amphibians and fish and the mesencephalicus lateralis, pars dorsalis (MLd) in birds, are important early processing centers for auditory signals produced in social interactions. Behaviorally, the auditory midbrain is necessary for responses to mate calls in some species (Endepols et al., 2003). Stimulus-evoked gene activity in the torus semicircularis reflects discrimination among different vocal signals, sometimes in a sex-specific manner (Hoke et al., 2004, 2010; Mangiamele and Burmeister, 2011). Furthermore, the processing of social vocalizations in auditory midbrain nuclei is context-dependent, in that neural responses vary with natural or experimental manipulation of reproductive state, or with salient factors such as the prospect of reward (Goense and Feng, 2005; Maney et al., 2006; Metzger et al., 2006; Miranda and Wilczynski, 2009a,b).

Consistent with these more global measures of the discrimination of social signals, individual IC neurons may themselves be selective for communication calls. With some exceptions, auditory midbrain neurons in a wide range of species show greater responses for auditory signals over nonvocalization sounds with similar characteristics (including time-reversed vocalizations), and may further be selective for specific vocalizations (Diekamp and Schneider, 1988; Bodnar and Bass, 1997; Crawford, 1997; Alder and Rose, 2000; Woolley et al., 2006; Andoni et al., 2007; Pincherli Castellanos et al., 2007; Suta et al., 2007, 2008; Andoni and Pollak, 2011; Elliott et al., 2011). In demonstrating selectivity for vocalizations, auditory midbrain neurons differ from neurons in some lower auditory nuclei. IC neurons show a higher level of vocalization selectivity than neurons that project to the IC from the dorsal and intermediate nuclei of the lateral lemniscus as a result of the convergence of inhibitory and excitatory inputs (Klug et al., 2002; Xie et al., 2005). Additional spectral or temporal coding mechanisms further contribute to selective midbrain responses (Epping, 1990; Bodnar and Bass, 1997; Leroy and Wenstrup, 2000; Bauer et al., 2002; Klug et al., 2002; Yavuzoglu et al., 2010; Andoni and Pollak, 2011; Pollak et al., 2011; Schneider and Woolley, 2011; Yavuzoglu et al., 2011), and patterns at the level of the neuron population may further increase the selectivity of vocalization encoding (Bodnar et al., 2001; Holmstrom et al., 2010; Schneider and Woolley, 2010).

Completing the link between serotonin and social situations, serotonin directly modifies the processing of social stimuli in the IC. In *in vivo* experiments in Mexican free-tailed bats, exogenously applied serotonin modulates the responses of individual IC neurons to playback of vocalizations from social interactions (Hurley and Pollak, 2005a). For most neurons, serotonin increases the selectivity of neural responses across a range of calls. Based on the regulation of less complex stimuli by different receptor types, reasonable predictions are that the modulation of vocalization responses by particular receptor types should vary among neurons, should depend in part on the structure of a given call, and that different serotonin receptor types may interact to influence vocalization coding in novel ways.

All of this information on serotonergic function in the IC at multiple levels of analysis suggests a possible scenario of the role of serotonin in social encounters. During social interactions, serotonin in the IC would steadily increase over the course of a social interaction, and would be related to the magnitude of the behavioral response. Many IC neurons would show a parallel increase in selectivity for vocalizations or other sounds produced during the encounter. Overall, this could create population-level responses for different vocalizations that are more distinct from each other in the presence of serotonin. This scenario serves as the basis for an as yet untested prediction that behavioral responses to social vocalizations correspond to levels of serotonin in the IC.

## Serotonin-auditory plasticity; a channel for past experience

**Figure 9:**
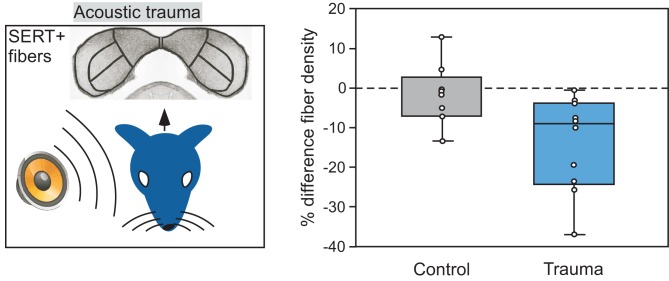
**Comparison of the density of serotonergic fibers in the IC contralateral versus ipsilateral to an ear exposed to 8 kHz acoustic trauma.** Left panel depicts the monaural trauma and subsequent immunolabeling of SERT-positive projections in the IC. Right panel shows percentage differences in fiber density in the IC contralateral to acoustic trauma relative to the IC ipsilateral to the trauma, in 10 CBA/J mice. Fiber density is lower in the contralateral relative to the ipsilateral IC in the trauma group, but not for the control group that did not receive trauma. The right panel was reprinted with permission from Elsevier; additional information on the source article can be found in the Acknowledgments.

An exciting prospect that has emerged as a result of work in the IC and other sensory regions is that plasticity in the infrastructure of the serotonergic system itself can provide a route for information about past events to influence neural circuitry. Plasticity in serotonergic-IC interactions occurs under a number of different conditions. The experience of acoustic trauma alters a fundamental aspect of the serotonergic system, the density of fibers within the IC. This is seen when a unilateral tonal trauma creates an imbalance in the density of serotonergic fibers between contralateral colliculi, with the IC contralateral to the traumatized ear having a lower density of fibers than the IC contralateral to the protected ear (Figure 9; Papesh and Hurley, 2012). Plasticity in the serotonergic system may also be guided by internal factors including age and reproductive state. Age influences both the density of serotonergic fibers and acute changes in serotonin, such that both fiber density and serotonin release during social encounters decline over young adulthood in mice (Hall et al., 2011; Papesh and Hurley, 2012). In older animals, serotonin content may rise again in the IC or other auditory regions (Cransac et al., 1996; Shim et al., 2012). Reproductive readiness, via gonadal hormones, also increases serotonergic fiber density in the avian auditory midbrain (Matragrano et al., 2012).

Plasticity is also observed at the level of serotonin receptors. With increasing age from adulthood through old age, the 5-HT2B receptor is upregulated in the IC (Tadros et al., 2007). Expression of this receptor type is further upregulated in old mice with severe hearing loss. The amount of expression of the 5-HT2B receptor is correlated with functional metrics of hearing such as the amplitude of the distortion product otoacoustic emission (DPOAE) and with the threshold of the ABR. In addition to age, peripheral damage leads to receptor plasticity. Following cochlear ablation, the expression of both 5-HT2C and 5-HT5B receptors is altered, but in different directions and with different dynamics. 5-HT2C expression increases from 3 through 90 days following ablation, and 5-HT5B receptor expression decreases from 5–21 days, and recovers by 90 days following ablation (Holt et al., 2005). Thus, the balance of different serotonin receptor types may be altered by plastic responses to aging or peripheral damage. Whether this creates qualitative changes in the modulatory effects of serotonin on auditory processing in the IC is unknown.

Predicting the functional importance of changes in the serotonergic infrastructure following events such as acoustic trauma is even more complex against the background of changes in other neurochemical signaling systems. Multiple events that create plasticity in the serotonergic system, such as acoustic trauma, are well-known for triggering plasticity in both excitatory and inhibitory neurotransmitter systems in the IC (Milbrandt et al., 2000; Dong et al., 2010; Browne et al., 2012). Such changes may result in shifts in excitatory-inhibitory balance in association with pathologies such as tinnitus and hyperacusis (Szczepaniak and Moller, 1996; Eggermont and Roberts, 2004). For example, experiments in the IC have demonstrated neurochemical and functional changes related to inhibition after acoustic trauma that have been associated with hyperexcitability of IC neurons (Salvi et al., 1990; Milbrandt et al., 2000; Salvi et al., 2000; Alvarado et al., 2005; Izquierdo et al., 2008; Browne et al., 2012). Given the evidence for serotonergic regulation of both inhibitory and excitatory neural responses in the IC, understanding exactly how events like acoustic trauma alter the regulation of excitability through particular receptor types is a topic that will require additional exploration.

Although plasticity at multiple levels is turning out to be a prominent feature of serotonergic-auditory interactions, whether particular types of changes represent developmental programs, homeostatic responses to peripheral damage, or pathological consequences of damage has not been addressed. Some insight into these issues may be gained from recent studies on the role of serotonin in visual plasticity. In rat visual cortex, ocular dominance plasticity, normally highly limited in adults relative to juveniles, is augmented by systemic administration of fluoxetine, a selective serotonin reuptake inhibitor (Maya Vetencourt et al., 2008). Other treatments that increase serotonin levels in the visual cortex, even behavioral treatments such as environmental enrichment, also augment ocular dominance plasticity (Baroncelli et al., 2010).

In light of this work, plasticity in the serotonergic system within the IC has several important implications for auditory function. One of these is based on the role of serotonin in mediating the influence of behavioral context. Plasticity in the serotonergic system could alter the relationship between behavioral context and auditory processing, leading to either more adaptive or pathological modulation of auditory responses during social interactions or stressful situations. If serotonin gates experience-dependent plasticity in the IC as it does in visual cortex, an alteration in the serotonergic system could additionally recast the ability of the auditory system to express adaptive plasticity. Both of these are exciting possibilities for future experimental attention.

## General conclusions

An increasing number of studies on the influence of serotonin in the IC have been completed in the last decade. Despite this, an understanding of serotonergic regulation in the IC, and indeed of the neuromodulation of auditory processing in general, is in its infancy. Here we have made the case that serotonin provides a channel for contextual information about internal state, external events, and even past experience. This information is then transformed into specific changes in the function of the neural circuits of the IC through parallel but interacting receptor pathways. Plasticity in the infrastructure of the serotonergic system, including within specific receptor pathways, could potentially alter the relationship between behavioral context and circuit function in important ways that have yet to be explored.

### Conflict of interest statement

The authors declare that the research was conducted in the absence of any commercial or financial relationships that could be construed as a potential conflict of interest.

## Abbreviations

8-OH-DPAT: 8-Hydroxy-2-(di-n-propylamino)tetralin
CP93129: 3-(1,2,3,6-tetrahydropyridin-4-yl)-1,4-dihydropyrrolo[3,2-b]pyridin-5-one
DNLL: dorsal nucleus of the lateral lemniscus
DOI: 2,5-dimethoxy-4-iodoamphetamine
DRN: nucleus raphe dorsalis
IC: inferior colliculus
MK212: 2-Chloro-6-(1-piperazinyl)pyrazine
mCPBG: m-chlorophenylbiguanidine
pCPA: parachlorophenylalanine
SERT: serotonin transporter
SSRI: selective serotonin reuptake inhibitor.
